# Complementary and Alternative Medicine in Oncology: A Concise Review of Utilization, Evidence, and Integration Challenges

**DOI:** 10.1002/cnr2.70509

**Published:** 2026-03-06

**Authors:** Danica Schöneseiffen, Matthias B. Stope

**Affiliations:** ^1^ Division for Physical Plasma Medicine, Department of Gynecology and Gynecological Oncology University Hospital Bonn Bonn Germany

**Keywords:** homeopathy, integrative medicine, oncology, patient well‐being and safety, phytotherapy, traditional Chinese medicine

## Abstract

**Background:**

Complementary and alternative medicine (CAM) is widely used by cancer patients and increasingly shapes oncological care worldwide. This narrative review examines current evidence on utilization patterns, theoretical frameworks, clinical efficacy, and safety aspects of CAM in oncology. CAM use is primarily motivated by emotional distress, the desire for personal autonomy, and perceived limitations of conventional cancer treatments. Despite its prevalence, communication about CAM between patients and physicians remains insufficient, and potential risks, particularly herb–drug interactions, are often underestimated in clinical practice.

**Recent Findings:**

Evidence indicates that selected CAM interventions, including acupuncture, ginger, and mind‐body therapies, provide measurable clinical benefits and are supported by plausible biological mechanisms. In contrast, the majority of CAM modalities are characterized by heterogeneous evidence bases that frequently rely on preclinical data or methodologically limited clinical studies. Persistent challenges such as publication bias, inconsistent outcome measures, and inadequate standardization substantially limit the interpretability and generalizability of existing findings. These limitations complicate evidence‐based decision making and hinder responsible clinical integration.

**Conclusion:**

Effective integration of CAM into oncology requires structured consultation models, systematic risk assessment strategies, and the inclusion of evidence‐based CAM education within medical training. In parallel, coordinated research initiatives and regulatory frameworks are essential to improve methodological rigor and ensure patient safety. This review concludes that only through scientific discipline, transparent evaluation processes, and interdisciplinary collaboration can CAM transition from a fragmented adjunct to a validated and ethically grounded component of comprehensive cancer care. Beyond summarizing existing evidence, the review highlights underrecognized mechanistic links and system‐level challenges that offer new perspectives on the future incorporation of CAM into modern oncology.

## 1 Introduction

The use of complementary and alternative medicine (CAM) among oncology patients is a globally observed phenomenon that has gained increasing relevance over recent decades. The motivations for turning to CAM are diverse and include a fundamental mistrust of pharmacological and chemical substances, as well as religious or spiritually motivated beliefs. Interest in alternative therapies rises particularly in the context of incurable diseases. Conventional treatments often impose substantial physical and emotional burdens and do not always yield the expected results [1, 2, 3, 4, 5].

A Europe‐wide study from 2005 found that 36% of surveyed cancer patients had used at least one form of CAM therapy [6].

Despite its prevalence, there remains no internationally standardized definition of CAM. The term is variably applied and often used synonymously, although it encompasses distinct conceptual approaches. According to the United States National Center for Complementary and Integrative Health (NCCIH), complementary medicine refers to therapeutic methods used in conjunction with conventional treatments, whereas alternative medicine replaces them entirely [7]. A combination of both approaches is known as integrative medicine, in which the patient is central and physical, psychological, social, and spiritual dimensions are incorporated into the therapeutic decision‐making process [8]. Teichfischer and Münstedt characterize alternative medicine as fundamentally incompatible with conventional medicine, while complementary medicine is regarded as potentially integrative within biomedical frameworks [9].

This conceptual progression from CAM to integrative oncology reflects a shift from isolated adjunctive therapies toward a structured, evidence‐informed incorporation of selected interventions within conventional cancer care. It emphasizes not only individual modalities but also their systematic integration into clinical practice.

## 2 Theoretical Foundations and Classification

The sheer diversity of CAM therapies complicates efforts at standardized classification. Conceptually, CAM encompasses interventions based on ingested or applied materials, such as the intake of biological products or the application of manipulative approaches, as well as those that rely solely on the patient's mental or physical engagement. The latter includes mind–body practices, modulating physiological states through cognitive, emotional, or behavioral techniques without the use of external agents.

The NCCIH distinguishes three primary groups (Table 1): natural products such as vitamins, minerals, or plant‐based remedies; mind–body practices including meditation, breathing exercises, yoga, and music therapy; and other modalities such as Traditional Chinese Medicine (TCM) or homeopathy [7].

**TABLE 1:** Classification by the National Center for Complementary and Integrative Health (NCCIH).

NCCIH group		Examples
1	Natural products	Vitamins, minerals, plant‐based remedies (phytotherapy)
2	Mind–body practices	Meditation, yoga, breathing exercises, music therapy
3	Other modalities	Traditional Chinese medicine (TCM), homeopathy

An alternative system categorizes CAM into four domains (Table 2). These include holistic medical systems based on closed theoretical constructs (e.g., TCM, homeopathy), mind–body therapies focusing on the interaction between mental and physical states, manipulative body therapies like massage or hyperthermia, and biological therapies, which overlap with the domain of natural products [10].

**TABLE 2:** Categories according to the S3 guideline on ‘Complementary Medicine in the Treatment of Oncology Patients’ (registration number: 032‐055OL).

Domain		Description	Examples
1	Holistic medical systems	Based on closed theoretical constructs	Traditional Chinese medicine (TCM), homeopathy
2	Mind–body therapies	Focus on the interaction between mental and physical states	Meditation, yoga, breathing exercises
3	Manipulative body therapies	Include physical manipulation methods (e.g., massage, hyperthermia)	Massage, Hyperthermia
4	Biological therapies	Overlap with the domain of natural products	Vitamins, Minerals, Herbal remedies

The two classifications differ primarily in structure and level of detail. The NCCIH system is clear and easy to understand but offers limited differentiation. In contrast, the S3 guideline provides a more precise scientific categorization. However, its structure is more complex and less intuitive for lay audiences. Both approaches acknowledge mind–body interventions as a central category. While the NCCIH system is advantageous for international communication and patient education, the S3 structure offers greater scientific precision. In practice, many therapies defy clear classification, posing challenges particularly for laypersons. Even medical professionals may encounter difficulties in categorizing CAM interventions due to their often multimodal nature [11].

## 3 Utilization and Distribution of CAM Therapies

Studies indicate wide variation in the use of CAM among cancer patients across different countries, with reported prevalence ranging from below 10% to over 70% [12]. CAM use is particularly common among female oncology patients, especially those with breast cancer or other gynecological malignancies [13, 14, 15]. The typical CAM user is often younger, female, more educated, and of higher socioeconomic status [16, 17, 18, 19]. Motivations are multifaceted and range from the desire to play an active role in treatment decisions to the alleviation of treatment‐related side effects, enhancement of immune function, and improvement of physical and psychological well‐being [20, 21, 22, 23, 24, 25, 26].

Psychological distress also appears to be a significant predictor of CAM use. Patients suffering from anxiety, emotional strain, or depression are especially inclined to explore complementary interventions [27, 28, 29]. This is particularly true for women diagnosed with gynecological cancers, who frequently report high levels of psychological burden [30, 31, 32, 33, 34]. Johnson et al. demonstrated that more than half of such patients experience substantial emotional stress [35]. Moreover, patients undergoing intensive treatment regimens and those occupying a passive role in treatment decisions are often confronted with increased stress levels and diminished quality of life [36, 37].

The economic burden associated with its use also plays a significant role in the decision to choose complementary medicine. In general, individuals, particularly in low‐income countries, but also in middle‐ and high‐income settings, experience a substantial financial burden during cancer treatment [38]. Various risk factors have been identified, including sociodemographic, socioeconomic, and medical factors, as well as systemic aspects related to health insurance [39]. With women and publicly insured patients being identified as the groups most severely affected [40].

In Germany, coverage of complementary medicine by public health insurance is highly restricted, encompassing only a limited number of CAM modalities. Even reimbursement through private insurers remains non‐standardized and varies according to individual policy provisions [41]. Particularly in resource‐limited countries, where the use of complementary medicine might also be deeply embedded in local cultural practices, financial costs and insufficient insurance coverage can represent a significant contributor to financial distress following a cancer diagnosis [42].

## 4 Sources of Information, Doctor–Patient Communication, and Associated Risks

Despite the widespread use of CAM, information about its application is predominantly obtained through non‐medical sources. The most frequently cited resources include family, friends, books, the internet, and magazines. Physicians and healthcare professionals are seldom the initial informants regarding CAM [43, 44, 45, 46]. In countries with higher CAM prevalence, patients are more likely to report medically supervised CAM use [47].

A major concern is the inadequate communication between cancer patients and their treating oncologists. In a study by Roberts et al., 61.1% of participants reported never having discussed their CAM use with a physician [48]. Similar trends are noted in other studies [49, 50, 51, 52]. Reasons for this lack of communication include patients' perceptions that CAM is irrelevant to conventional treatment or fears of disapproval by their physicians [53]. While most doctors do not fundamentally reject CAM, they frequently emphasize safety considerations without offering a nuanced assessment of efficacy [54].

This communication gap poses serious risks. Many patients believe that natural products are inherently harmless, underestimating the potential for pharmacodynamic and pharmacokinetic interactions [55, 56, 57]. These interactions are especially pertinent to drugs metabolized via the cytochrome P450 enzyme system. Enzyme‐induced changes can lead either to diminished drug efficacy or to increased toxicity, both of which are critical concerns in the context of chemotherapy or targeted therapies [58, 59, 60].

## 5 Efficacy, Mechanistic Insights, and Safety of CAM

A central limitation in the scientific appraisal of CAM lies in the paucity of high‐quality clinical data. Existing studies often suffer from small sample sizes, methodological heterogeneity, or bias. Nonetheless, some individual studies report beneficial outcomes. For instance, acupuncture has been shown to effectively reduce nausea and pain in several trials [61, 62], and ginger has demonstrated prophylactic efficacy in managing chemotherapy‐induced nausea [63, 64]. Zinc supplementation appears to help prevent mucositis [65], and enzymes like bromelain have been proposed to potentiate the cytotoxic effects of chemotherapeutic agents [66, 67]. Furthermore, movement‐based therapies such as yoga have been associated with improvements in psychosocial factors like stress reduction and overall quality of life [68, 69]. Despite these promising findings, the current evidence remains insufficient to support the broad implementation of CAM as a standard evidence‐based practice. Ethical concerns arise when comprehensive risk–benefit assessments cannot be reliably conducted [70, 71, 72, 73].

In addition to clinical evidence, an increasing body of molecular and cellular data provides insight into the biological mechanisms of various CAM approaches. For acupuncture and electroacupuncture, modulation of central signaling cascades such as NF‐κB, MAPK, and JAK/STAT has been demonstrated, affecting cytokine release, T‐cell differentiation, and natural killer cell activity [74]. Mind–body interventions such as meditation and yoga induce measurable transcriptomic changes in leukocytes. These include downregulation of proinflammatory NF‐κB‐driven gene programs and activation of interferon‐dependent antiviral pathways [75, 76]. The so‐called “relaxation response” further induces genome‐wide changes in energy metabolism, insulin signaling, and inflammatory networks [77].

Physical manipulation‐based therapies such as hyperthermia can potentiate radiotherapy. They enhance blood flow and vascular permeability, promote tumor reoxygenation, and increase the radiosensitivity of otherwise resistant malignant cells [78].

Phytotherapeutic compounds have also been investigated in detail. Curcumin interferes with NF‐κB, STAT3, and COX‐2 pathways, thereby inhibiting proliferation and inducing apoptosis in various tumor models [79]. Epigallocatechin gallate, the main catechin of green tea, modulates PI3K/AKT, NF‐κB, and angiogenic signaling pathways, resulting in antiproliferative and pro‐apoptotic effects [80]. For ginger and its bioactive component 6‐gingerol, inhibition of COX‐2 and NF‐κB has been shown, along with reduced tumor cell migration and induction of apoptosis [81]. Extracts and lectins from mistletoe ( *Viscum album* ) have been reported to induce apoptosis via caspase‐8 and ‐9 activation, cause cell cycle arrest, and enhance NK cell cytotoxicity [82]. These studies demonstrate that diverse CAM approaches target defined molecular structures and cellular processes. They modulate stress and survival pathways, immune cell function, and tumor cell proliferation and apoptosis. Such mechanistic insights strengthen the biological plausibility of CAM and underscore its translational potential in oncology.

Safety concerns related to CAM interventions are of particular importance in oncology, where multimodal regimens are common. Herb–drug interactions can significantly alter pharmacokinetics or pharmacodynamics of anticancer agents. A well‐documented example is St. johannis herb ( *Hypericum perforatum* ), which induces cytochrome P450 3A4 (CYP3A4) and P‐glycoprotein. Co‐administration has been shown to reduce exposure to irinotecan and imatinib, with increased clearance in clinical pharmacokinetic studies [83, 84]. For taxanes, a clinical study demonstrated decreased docetaxel exposure under St. johannis herb pre‐supplementation. Collectively, these data support routine medication reconciliation and explicit counseling on herbal products. In addition, antioxidant supplementation during radiation therapy and some chemotherapy regimens remains controversial. Authoritative reviews and randomized trials in head and neck cancer have raised concerns that high‐dose antioxidant vitamins may adversely affect recurrence or survival, warranting caution and individualized risk–benefit assessment [83, 85, 86, 87]. Beyond the potential adverse effects associated with their intrinsic biological activity, herbal medicinal products also pose quality‐control risks. They may be adulterated with toxic heavy metals or prescription pharmaceuticals, contaminated with microorganisms such as fungi or bacteria, and frequently exhibit insufficient product standardization and inadequate labeling [88, 89]. Table 3 provides a concise synthesis of the most frequently investigated CAM modalities in oncology, highlighting their therapeutic applications, best available evidence, and associated safety concerns.

**TABLE 3:** Summary of selected CAM interventions in oncology.

Intervention	Indication/application	Best available study design (as cited)	Main reported effect	Key safety aspect
Acupuncture	CINV, cancer pain	RCTs/systematic reviews [61, 62]	↓ nausea and pain, ↑ symptom control	Well tolerated; requires trained practitioners
Ginger ( *Zingiber officinale* )	CT‐induced N/V	Meta‐analysis of RCTs [63, 64]	Prophylactic antiemetic effect, improved tolerance of CT	Possible mild GI discomfort; potential PK interactions require evaluation
Zinc	Prevention of mucositis	RCT [65]	↓ incidence/severity of mucositis	High doses → copper imbalance; ↓ toxicity at recommended doses
Bromelain (enzyme therapy)	Supportive treatment in solid tumors/breast cancer models	Preclinical studies (in vitro+in vivo) [66, 67]	↑ cytotoxic effect of CT; enhanced antitumor activity	Theoretical risk of GI bleeding/irritation; limited human safety data
Yoga/mind–body therapy	Psychological distress, fatigue, QoL	Controlled clinical trials/systematic reviews [66, 67]	↑ stress resilience, mood and QoL during therapy	Minimal risk; tailor to mobility of patient
Meditation/Relaxation response	Stress modulation, immune regulation	RCTs with molecular endpoints [75, 76, 77]	↓ NF‐κB; ↑ IFN pathways	No direct adverse effects reported; psychological readiness required
Hyperthermia (physical therapy)	Adjunct to RT/CT	Mechanistic+clinical adjunct studies [78]	↑ tumor perfusion, ↑ radiosensitivity; potential synergy with cytotoxic therapy	Requires temperature control; risk of tissue overheating
Curcumin ( *Curcuma longa* )	Experimental anticancer phytotherapy	Molecular/preclinical studies [79]	Inhibition of NF‐κB, STAT3, COX‐2; pro‐apoptotic/anti‐proliferative effects	Variable bioavailability; possible interference with drug metabolism
Epigallocatechin gallate (green tea catechin)	Experimental adjunctive therapy	Molecular/preclinical studies [80]	Modulation of PI3K/AKT, NF‐κB, angiogenic signaling; antiproliferative effects	Risk of high‐dose hepatotoxicity; may affect drug absorption
Gingerol (active compound of ginger)	Experimental cytotoxic and anti‐inflammatory	Molecular/cellular studies [81]	COX‐2 and NF‐κB inhibition; ↓ tumor migration; apoptosis induction	Generally safe at dietary doses; caution at pharmacological concentrations
Mistletoe extract ( *Viscum album* L.)	Adjunctive therapy in hematologic and solid malignancies	In vivo AML model/cell culture [82]	Induction of apoptosis via caspase‐8/‐9, NK‐cell activation	Possible allergic reactions; product standardization and contamination risk
Probiotics	Prevention of mucosal and GI side effects	Clinical observational/interventional studies [70, 71, 72]	May support mucosal integrity and immune modulation	Infection risk in immunocompromised patients; strain selection critical
St. John's wort ( *Hypericum perforatum* )	Herbal antidepressant used concomitantly with CT	Clinical pharmacokinetic studies [83, 84, 85, 86]	CYP3A4 and P‐gp induction → ↓ exposure to irinotecan, imatinib, docetaxel	Major risk of reduced efficacy of anticancer agents; contraindicated co‐use
High‐dose antioxidant vitamins	Support during RT/CT	RCTs/reviews [85, 86, 87]	No consistent survival benefit; possible adverse influence on recurrence	Potentially harmful in high doses; should be individualized
Traditional Chinese Medicine (TCM) herbs	CT side‐effect management (colorectal cancer etc.)	Cochrane systematic review [71]	Some symptom relief reported (heterogenous)	Quality control and contamination issues; herb–drug interactions possible

*Note:* Clinical Evidence and Safety.

## 6 Providers and Institutional Frameworks

The spectrum of CAM providers is diverse. In addition to physicians, practitioners include nonmedical professionals such as naturopaths, nurses, physical therapists, and others. In Germany, the number of physicians with additional qualifications in CAM increased from approximately 40 000 in 2005 to around 65 000 in 2020, with acupuncture representing a particularly common specialty [90]. General practitioners and orthopedic specialists are among the most CAM‐affine medical groups [91, 92]. Surveys reveal generally positive attitudes among physicians, though many report uncertainties regarding application and patient counseling. Only a minority possess formal training, though there is widespread interest in integrating CAM more effectively into medical education [93, 94, 95, 96, 97, 98, 99].

The role of nonphysician CAM providers, especially naturopaths, is particularly controversial. Many patients consult them seeking more personalized care and holistic attention. Time, in particular, is a crucial factor in enabling patients to feel acknowledged. Nonphysician healthcare professionals are often able to provide more time‐intensive care, whereas physicians frequently have only limited time up to a few minutes to engage meaningfully with their patients [100, 101, 102]. However, significant deficiencies exist in training quality and regulatory oversight. Research indicates that the majority of naturopathy schools do not meet recommended educational standards [103]. In oncology, this poses potential risks, especially when CAM is misinterpreted as a replacement for evidence‐based treatment [104].

Interdisciplinary collaboration between physicians and nonmedical CAM practitioners is generally accepted when the limits of complementary interventions are respected and no interference with conventional therapies occurs [105]. To promote safe and competent CAM care, particularly for cancer patients, criteria were published in 2020 to help patients distinguish between reputable and questionable providers [106]. According to these criteria, a reputable provider of complementary medical services should inquire about the patient's individual diagnosis and current treatments, openly address potential interactions with oncological therapy, and justify the recommended intervention in an evidence‐based and patient‐specific manner. The possibilities and limitations, as well as the goals, content, duration, and costs, should be presented transparently. In addition, the provider should allow the patient sufficient time for consideration.

## 7 Limitations and Implications

This review provides a narrative synthesis of current evidence on CAM in oncology. Several methodological limitations must be considered when interpreting the findings.

First, the heterogeneity of CAM definitions and study designs complicates direct comparison between trials and may obscure intervention‐specific effects. Variability in outcome parameters, patient populations, and reporting quality further limits the ability to generalize results across settings. Second, selection and publication bias are inherent challenges in this field, as studies reporting positive outcomes are more likely to be published, while neutral or negative findings often remain underrepresented. The lack of standardized evaluation frameworks and consistent quality control further contributes to the uneven evidence landscape. Furthermore, most data originate from single centers or highly specific cancer populations, which constrains external validity and transferability to broader oncological practice. Moreover, differences in healthcare systems, reimbursement structures, and cultural attitudes toward CAM impede cross‐country comparability.

Despite these limitations, the synthesis highlights key clinical and systemic implications. Integrative oncology requires the development of structured frameworks for risk assessment, patient counselling, and interdisciplinary collaboration. Clear communication between patients, oncologists, and CAM providers is crucial to minimize risks and to ensure evidence‐based, patient‐centered decision‐making.

## 8 Advancing Integrative Oncology: Clinical, Educational, and Policy Directions

Building upon the identified gaps and implications, the advancement of integrative oncology depends on coordinated efforts that align research quality, clinical governance, and professional education.

Research priorities should include rigorously designed randomized and translational studies that verify mechanistic plausibility and evaluate clinically meaningful outcomes. Transparent reporting, harmonized endpoints, and systematic inclusion of negative findings are essential to strengthen scientific reliability and reproducibility. In clinical practice, structured CAM consultation services within oncology departments can provide a supervised framework for patient assessment, documentation, and counselling. Such institutionalized services facilitate safe therapeutic integration, promote interprofessional exchange, and enhance transparency for both patients and healthcare providers.

Education and training represent key enablers for responsible CAM integration. The inclusion of evidence‐based CAM modules in medical and nursing curricula would enhance professional competence and reduce misinformation.

Finally, health policy must establish clear regulatory and quality assurance standards, particularly for nonphysician CAM providers, to safeguard patient welfare and professional accountability.

Taken together, advancing integrative oncology requires a unified strategy that embeds scientific rigor, patient safety, and interdisciplinary collaboration into all levels of oncological care, transforming CAM from a fragmented adjunct into a transparent, evidence‐supported dimension of modern cancer medicine.

## 9 Conclusion

CAM has become an integral yet still controversial aspect of modern oncology. The persistent interest among patients reflects an unmet need for holistic, patient‐centered approaches that extend beyond symptom control and address psychological, social, and existential dimensions of cancer care. However, this growing utilization also reveals substantial challenges regarding evidence quality, communication, and regulatory oversight.

Current research demonstrates that only a limited subset of CAM interventions, such as acupuncture, ginger, and mind–body therapies, demonstrates reproducible clinical benefit supported by mechanistic plausibility. These examples illustrate the potential of integrative approaches when they are implemented within a framework of scientific rigor and medical supervision. In contrast, the evidence base for many phytotherapeutic and supplement‐based interventions remains inconsistent, often derived from preclinical data or small, methodologically heterogeneous clinical studies. Publication bias, limited sample sizes, and the frequent absence of standardized endpoints continue to impede reliable interpretation and meta‐analytic synthesis.

From a clinical perspective, the uncontrolled use of unverified CAM therapies poses tangible risks through pharmacokinetic interactions, contamination, and delayed initiation of evidence‐based oncological treatment. Accordingly, structured CAM consultation services within oncology institutions represent a crucial safeguard. Such services can provide systematic risk assessment, document ongoing use, and ensure informed counselling. They also facilitate transparent communication among patients, oncologists, and nonmedical practitioners, representing an essential prerequisite for patient safety and shared decision‐making. Integrating CAM into structured clinical pathways therefore increases patient safety by systematically identifying potential herb–drug interactions and other contraindications that frequently remain unrecognized in routine care. Such integration enables oncologists to evaluate complementary therapies within the full clinical context, anticipate risks earlier, and prevent avoidable treatment complications.

Future progress depends on coordinated efforts across research, education, and policy. Methodologically robust clinical trials, harmonized reporting standards, and translational studies linking molecular mechanisms to patient outcomes will be decisive in establishing scientific validity. Although the current evidence base limits the extent to which CAM‐related content can already be integrated into medical curricula, expanding education in parallel with advances in research is crucial. Improved training will help future oncologists recognize the breadth of complementary practices their patients may already be using, often without disclosure, and proactively address them, thereby narrowing the persistent knowledge gap between clinicians and patients.

Finally, clear regulatory frameworks and quality assurance mechanisms must enforce accountability and consistency across providers. In addition to promoting professional accountability, such regulatory structures are essential for safeguarding patient safety, particularly in light of the heterogeneous training backgrounds of CAM providers and the variable quality of biologically based products. Strengthening oversight mechanisms that address practitioner competence, documentation standards, and product safety can help prevent avoidable harm and ensure that patients are protected when using complementary interventions. Beyond these clinical and regulatory considerations, integrative oncology may also provide meaningful economic and social benefits. A recent systematic review reported that certain complementary and integrative therapies were associated with reduced healthcare utilization in oncology suggesting potential efficient resource allocation [107]. In addition, psychosocial studies indicate that integrative oncology programs can enhance patients' emotional well‐being, coping capacity, and overall quality of life, reflecting broader social benefits that further support the development of structured integrative care models [108].

**FIGURE 1:**
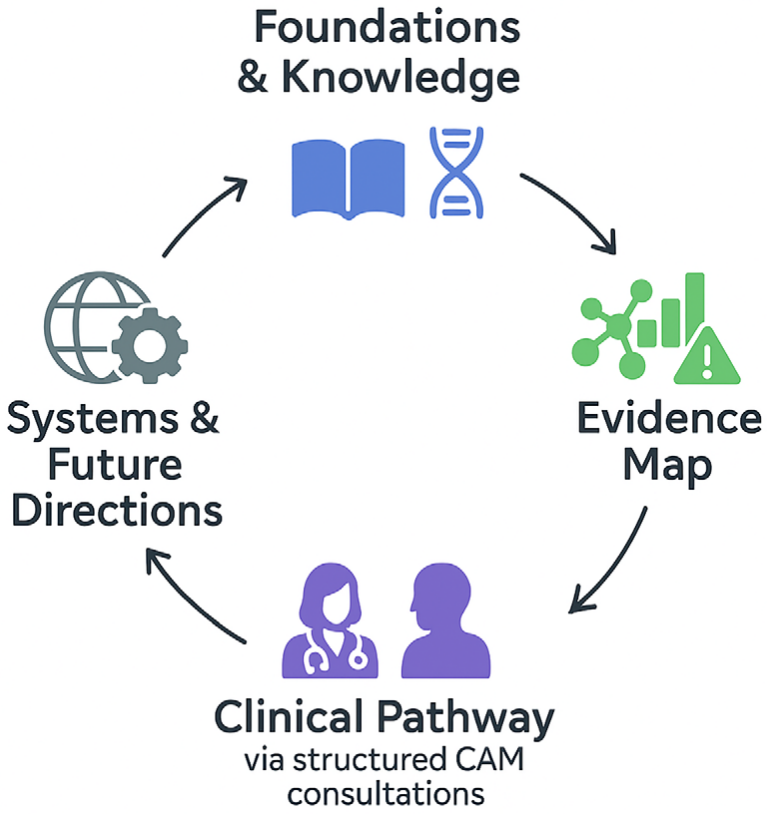
Progressive and evidence‐based integration of CAM in oncology, linking *Foundations & Knowledge* and the *Evidence Map* to clinically supervised application within the *Clinical Pathway*. This process advances toward sustainable implementation through *Systems & Future Directions*, ensuring methodological rigor, patient safety, and translational continuity. *Source:* Figure created by the authors.

In conclusion, advancing integrative oncology requires a deliberate balance between open‐mindedness and scientific discipline. By embedding CAM within evidence‐based, ethically guided, and professionally supervised care structures, oncology can transform fragmented empirical practices into a coherent, transparent, and patient‐centered model of comprehensive cancer medicine (Figure 1).

## Author Contributions

**Danica Schöneseiffen:** conceptualization, writing – original draft, visualization, writing – review and editing. **Matthias B. Stope:** conceptualization, writing – original draft, visualization, writing – review and editing, supervision.

## Funding

The authors have nothing to report.

## Ethics Statement

This article does not contain any studies with human or animal participants.

## Consent

The authors have nothing to report.

## Conflicts of Interest

The authors declare no conflicts of interest.

## Data Availability

Data sharing not applicable to this article as no datasets were generated or analysed during the current study.
